# Longitudinal formative assessment reforms in manual therapy education: impacts on psychological wellbeing and course satisfaction

**DOI:** 10.3389/fmed.2026.1823631

**Published:** 2026-06-26

**Authors:** Ruike Zhang, Wanqi Wu, Yuanxing Huang, Yongying Li, Yiqi Yang, Minglei Huang, Adila Aisaiti, Jinyang Xie, Rui Huang, Jiaqiao Yan, Kai Shi, Shuangquan Ji, Tao Huang, Zhenfeng Guo, Qinglu Luo

**Affiliations:** 1 Key Laboratory of Biological Targeting Diagnosis, Department of Rehabilitation Medicine, Therapy and Rehabilitation of Guangdong Higher Education Institutes, The Fifth Affiliated Hospital of Guangzhou Medical University, Guangzhou, China; 2 The Fifth Clinical College of Guangzhou Medical University, Guangzhou, China

**Keywords:** course satisfaction, education, formative assessment, manual therapy, psychological wellbeing

## Abstract

**Background:**

Traditional assessment (TA) model in medical education, especially in practice-intensive disciplines such as manual therapy, frequently intensify student stress and neglect psychological wellbeing, underscoring the necessity for creative evaluation strategies. Formative assessment (FA) provides a process-oriented methodology, although its psychological advantages are still insufficiently examined. The study aimed to investigate synergistic effects of FA on students’ psychological wellbeing, academic performance, and learning experience in manual therapy courses.

**Methods:**

This study included a total of 88 third-year physical therapy (PT) students. Groups B, C, and D constituted the prospective arms, while group A served as a historical control. Using standardized instruments (PHQ-9, GAD-7, CEQ), we assessed the impact of FA on psychological wellbeing, academic performance, and course satisfaction in comparison to TA across four groups. FA encompassed high-frequency, low-stakes tests and clinically focused evaluation criteria, characterized by variations in time and complexity.

**Results:**

FA significantly decreased anxiety socres (*F* = 2.965, *P* = 0.037) and improved course satisfaction scores (*F* = 11.306, *P* < 0.001), with the group D attaining the maximum satisfaction (95.30 ± 6.91) owing to prolonged preparation time and refined assessment schedule. The experimental groups exhibited diminished practical performance scores (*P* < 0.001), which can be ascribed to a paradigm shift toward stringent, clinically oriented rubrics rather than a decline in skill mastery. No notable variations were detected in depression levels (*F* = 1.565, *P* = 0.204) or overall academic performance (*P* > 0.05).

**Conclusion:**

FA facilitated the establishment of supportive, process-oriented learning environments in manual therapy education, correlating with reduced anxiety and improved course satisfaction while mitigating the limits of TA. The convergence of strategic task design and psychological support appears essential for maximizing the potential benefits of FA.

## 1 Background

The inherent challenges of medical education, including a demanding curriculum requiring extensive memorization, simultaneous evaluation of theoretical knowledge and practical skills, and rigorous scheduling (1–3), create significant systemic stressors. These pressures are intensified by fierce competition in the job market. At the end of 2023, China had 12.488 million health technical personnel (including physical therapy (PT) positions) (4). Medical job applications increased by 23% year-on-year, postgraduate-level positions rose by 15%, and the number of health technical personnel grew by 830,000 compared with the previous year (4–6). But the employment rate for master’s graduates was only 58.21% (7). Research indicates significant mental health issues among Chinese medical and health-related students, with a depression prevalence of 29% and an anxiety prevalence of 21% among (8, 9), highlighting a latent crisis within this high-pressure educational environment (10).

In China, PT is an independent undergraduate major within rehabilitation medical education, with graduates working as health technical personnel who are regarded as part of the broader medical education system (11). Consequently, PT students face systemic issues similar to those previously mentioned, particularly in practice-intensive courses such as manual therapy, where the profound integration of theory and practice exposes significant deficiencies in traditional assessment (TA) models (12). Although TA methods facilitate standardized implementation and enhance student engagement in assessments (13), their shortcomings are increasingly apparent. First, The TA model often leads to knowledge fragmentation, highlighting a significant disconnect between theory and practice (14). Second, intensive end-of-term evaluations exacerbate students’ psychological stress (15). Additionally, the lack of continuous feedback mechanisms impedes instructional improvement. Against the backdrop of surging demand for rehabilitation professionals (16), these issues underscore the urgent need for innovative reforms in educational assessment systems.

In response to these challenges, formative assessment (FA) arises as a potential educational technique that prioritizes process-oriented feedback (17, 18). FA in medical education, through high-frequency, low-stakes “assess-feedback-improve” cycles, provides numerous advantages: increased learning motivation, improved course mastery, enhanced practical competence, better student experience, and real-time monitoring for dynamic teaching adjustments (19, 20). Nonetheless, current research predominantly emphasizes academic achievement, skill development, and satisfaction indicators. For instance, undergraduate anatomy courses utilizing weekly Canvas quizzes with immediate feedback workshops significantly improved exam scores (particularly on clinical application questions) and learning confidence (17). Nursing clinical courses employing virtual simulation and real-time feedback with peer evaluation demonstrated enhanced clinical competence and learning experience through competency assessments and satisfaction surveys (21). Oral surgery courses implementing Mini Clinical Evaluation Exercise (Mini-CEX)/Direct Observation of Procedural Skills (DOPS) immediate feedback achieved superior summative exam performance and clinical decision-making abilities (22). Although these studies validate the academic advantages of FA, they predominantly neglect its immediate psychological effects (e.g., stress, anxiety), regarding mental health outcomes as ancillary and failing to directly quantify alterations in students’ psychological states. Furthermore, its implementation necessitates three essential conditions: process-oriented learning, low-stress environments, and regular FA (13, 23). Consequently, ongoing enhancement of evaluation methodologies and feedback systems is crucial for thorough educational reform.

To address these research gaps, this prospective cohort study, incorporating a historical control group, employs standardized psychological assessment tools (Patient Health Questionnaire, PHQ-9; Generalized Anxiety Disorder-7, GAD-7) to quantify mental health symptoms (8), combined with the Course Experience Questionnaire (CEQ) for teaching feedback evaluation (24). By innovatively integrating formative assessment (FA) with multidimensional educational outcome indicators, this longitudinal design systematically investigate its synergistic effects on students’ psychological wellbeing, academic performance, and learning experience in manual therapy courses. This approach provides empirical evidence for developing psychologically supportive practical teaching models aligned with student-centered educational principles.

## 2 Materials and methods

### 2.1 Participants and study design

This study employed a prospective cohort design with a historical control to compare the effects of four instructional methods in a physical therapy (PT) course. Conducted from March 2021 to July 2024, the study initially enrolled 97 PT students from the 2019 to 2022 cohorts at the at the Fifth Clinical College of Guangzhou Medical University (GZMU). All participants included in the study provided informed consent before course commencement. The study was approved by the Ethics Committee of the Fifth Affiliated Hospital of GZMU (GYWY-L2022-53) and conducted in strict accordance with the Declaration of Helsinki.

Inclusion criteria were: (1) third-year students from the 2019 to 2022 cohorts at the Fifth Clinical College of GZMU; (2) voluntary participation with consent to complete all questionnaires. Exclusion criteria were: (1) refusal to sign informed consent; (2) Physical discomfort or psychological assessment suggesting non-participation; (3) Incomplete theoretical exams or scores < 60 points.

A total of 88 students (90.7%) completed the study protocol and were included in the analysis. Nine students (9.3%) were excluded: 5 (5.2%) for incomplete post-course questionnaires (primarily due to internship conflicts or medical leave), 3 (3.1%) for withdrawing consent, and one (1.0%) was advised to undergo psychological intervention. Participants were divided by cohort into four groups: historical control group A (2019 cohort, *n* = 24, male/female = 8/16, age 19.29 ± 0.69), experimental group B (2020 cohort, *n* = 19, male/female = 6/16, age 19.27 ± 0.46), experimental group C (2021 cohort, *n* = 19, male/female = 7/12, age 19.42 ± 0.51), and experimental group D (2022 cohort, *n* = 23, male/female = 6/17, age 19.17 ± 0.57). Statistical analysis revealed no significant differences among groups in age (*p* = 0.585), gender distribution (*p* = 0.857), pre-course GAD-7 scores (*p* = 0.662), pre-course PHQ-9 scores (*p* = 0.840), or Functional Anatomy course grades (*p* > 0.05) (Tables 1, 2), indicating comparable baseline characteristics.

**TABLE 1:** Baseline comparison of demographic and psychological variables across four groups [*n* (%), M(P25, P75)].

Variables	Group A	Group B	Group C	Group D	χ^2^/H	*P*
Male	8 (33.3%)	6 (27.3%)	7 (36.8%)	6 (26.1%)	χ^2^ = 0.77	0.857
Female	16 (66.7%)	16 (72.7%)	12 (63.2%)	17 (73.9%)		
Age	19 (19, 20)	19 (19, 20)	19 (19, 20)	19 (19, 20)	H = 1.94	0.585
Depression (PHQ-9)	3 (3, 4)	3 (3, 4)	3 (3, 4)	3 (2, 4)	H = 0.84	0.840
Anxiety (GAD-7)	3 (2, 3)	3 (2, 3)	3 (1, 3)	2 (2, 3)	H = 1.59	0.662

Data are presented as count (percentage) or median (P25, P75). Comparisons among multiple groups for categorical variables (sex) were performed using the chi-square test. Comparisons among multiple groups (age, PHQ-9, GAD-7) were performed using the Kruskal-Wallis H test.

**TABLE 2:** Baseline comparison of academic performance across four groups [M(P25, P75)].

Variable	Group				*H*	*P*
	Group A	Group B	Group C	Group D		
Class performance	93.50 (91.00, 95.75)	96.00 (91.75, 97.00)	93.00 (91.00, 95.00)	93.00 (91.00, 95.00)	3.645	0.302
Practical performance	93.00 (90.00, 97.00)	98.00 (91.50, 98.00)	94.00 (92.00, 96.00)	91.25 (89.25, 95.00)	5.798	0.122
Theoretical score	74.00 (69.00, 79.75)	77.00 (70.50, 81.00)	76.00 (70.00, 81.00)	73.00 (70.00, 79.00)	1.697	0.638
Overall grade	81.00 (79.00, 85.75)	83.00 (78.75, 87.00)	83.00 (79.00, 86.00)	83.00 (80.00, 87.00)	1.187	0.756

Data are presented as median (P25, P75). Comparisons among multiple groups were performed using the Kruskal-Wallis H test.

### 2.2 Manual therapy course

Based on evidence-based medicine, the curriculum has seven clinically applicable modules: McKenzie Method (2 theory + 4 practical hours) for mechanical dysfunction; Fascial manipulation (2 theory + 8 practical) for fascial biomechanics in pain modulation and chronic pain management; Stretching Techniques (2 theory + 8 practical) for soft tissue extensibility and joint mobility; Neural mobilization for reducing neural tension-related dysfunction (2 theory + 8 practice); Lymphatic Massage (2 theory + 8 practical) promotes lymphatic drainage and metabolic function; Maitland Joint Mobilization (1 theory + 12 practical) uses rhythmic passive movements for pain relief and functional restoration; and Mulligan technique (2 theory + 8 practical) involves mobilization and active movement for immediate pain relief and functional reconstruction. Students completed Functional Anatomy as a prerequisite. All four groups completed an identical curriculum totaling 69 contact hours (where 1 contact hour = 40 min), consisting of 13 h of theoretical instruction and 56 h of practical training.

### 2.3 Teaching methods

#### 2.3.1 2019 cohort: historical control group A

Group A students adopt a “theoretical lecture + practical training” TA model. The course grade includes theoretical score (final written examination, 60%), practical performance (including final practical assessment (FPA), 30%), and class performance (10%). The FPA at the end of the course evaluates students’ practical skills across all chapters. Teachers post Power Point presentations (PPTs), short movies, and reference materials to the GZMU e-Learning Center for students to preview. Theoretical instruction primarily include lectures and Q&A sessions with online homework to enhance knowledge. Practical training uses “teacher demonstration-student practice-summary feedback.” In the first 30 min of class, the instructor spends 30 min demonstrating core knowledge points and procedures (e.g., grading standards for joint mobilization). This is followed by detailed demonstrations of techniques for specific body parts, after which students practice in groups. The session concludes with the instructor summarizing and providing feedback on the overall practice.

#### 2.3.2 2020 cohort: experimental group B

Group B adjusted the overall course grade structure to: 50% theoretical score, 40% practical performance (including both FPA and formative assessments (FAs), and 10% class performance. The weight of the FPA was reduced from 30 to 20%, and FAs for seven chapters (20%) were introduced. These FAs are conducted in the last 40 min of the second lab session of each chapter, involving 6–7 students, with other students and two instructors jointly grading. The FA score is a weighted average of student and instructor evaluations. The FPA follows the same format as group A, covering seven chapters with five questions per chapter, randomly selecting two for assessment. This design provides phased, continuous feedback to help students identify and address weaknesses promptly, reduce psychological stress from the FPA, enhance skill mastery, and boost confidence.

#### 2.3.3 2021 cohort: experimental group C

Group C shares the same overall grade structure as group B, with identical weightings for the FPA and FAs. However, based on the group B’s course feedback, group C introduced a comprehensive clinical problem-oriented evaluation in addition to the group B’s practical assessment questions (including both FPA and FAs). Feedback indicated that students need not only standardized operational skills but also the ability to handle complex clinical scenarios. Thus, this evaluation assesses not only the standardization of techniques but also clinical decision-making processes, including history-taking, physical sign evaluation, and selecting appropriate treatment methods. This approach simulates real clinical workflows to integrate students’ clinical thinking and operational skills, enhancing professional competence and alleviating psychological pressure from purely test-oriented assessments.

#### 2.3.4 2022 cohort: experimental group D

Group D modified group C’s assessment plan by reverting to group B’s unified assessment questions and replacing the FPA with FAs, which now account for 40% of the total grade. Additionally, the timing of FAs for each chapter was adjusted to the first 40 min of the lab session of the subsequent chapter, with grading standards and requirements consistent with group B. This design aims to provide students with more review and preparation time, reducing tension and anxiety caused by time constraints.

One teaching assistant left group C and one teacher left group D, but the teaching teams remained mostly the same. All four groups took 100-point final theoretical exams with the same difficulty, question styles, and scoring standards. Practical assessment rating varied greatly. The FPA for group A used typical scoring rubrics (Supplementary File 1) to assess procedural completeness and technical skill. Groups B, C, and D (both FA and FPA) used newly designed formative evaluation rubrics (Supplementary File 2) to bridge classroom instruction and clinical practice, a paradigm shift from “procedural assessment” to “comprehensive clinical competency evaluation.” Three important developments in the new rubrics reorganized evaluation parameters and placed greater focus on overall clinical abilities than conventional standards: (1) Clinicalized evaluation dimensions including “therapist-patient positioning,” “communication skills” (including procedural explanation, consent acquisition, and emotional support), and “humanistic qualities” (privacy respect and compassionate care); (2) The evaluation process is refined, breaking down “technique execution” into sub-items (location, force, frequency, duration, repetitions), guiding focus on operational quality rather than mere process replication. (3) The evaluation is competency-orientated, emphasizing the overall ability to perform operations safely, effectively, and patient-centeredly in simulated scenarios. New standards were more stringent and complicated, assessing clinical competency more thoroughly. The comparison of practical assessment designs across four groups (2019–2022) can be seen in Table 3.

**TABLE 3:** Comparison of practical assessment designs across four groups (2019–2022).

Group	Assessment type	Practical score composition	Assessment time	Key features
Group A	Traditional assessment	30% FPA	End-of-course only	Traditional rubric; emphasis on procedure completeness; no formative assessment
Group B	Formative and summative assessment	20% FPA and 20% FAs	Last 40 min of the 2nd lab session per chapter	Standard skills assessment; teacher and peer grading
Group C	Formative and summative assessment	20% FPA and 20% FAs	Same as group B	Added clinical problem-oriented evaluation; focuses on clinical reasoning and decision-making
Group D	Pure formative assessment	40% FAs (no FPA)	First 40 min of the 1st lab session of the next chapter	Extended preparation time; highest FA weight

FPA, Final Practical Assessment; FAs, Formative Assessment.

### 2.4 Evaluation

#### 2.4.1 Academic performance

Academic performance comprised three components: class performance, practical performance, and theoretical score. Class performance was comprehensively evaluated based on students’ level of engagement in classroom activities, including attendance, frequency of participation in discussions and Q&A sessions, and performance on regular assignments. Overall grades were calculated using the following group-specific weightings:

Group A: Overall grade = 10% class performance + 30% practical performance (30% FPA) + 60% theoretical score;

Groups B and C: Overall grade = 10% class performance + 40% practical performance (20% FPA + 20% FAs) + 50% theoretical score;

Group D: Overall grade = 10% class performance + 40% practical performance (40% FAs) + 50% theoretical score.

#### 2.4.2 Questionnaires

The PHQ-9, developed by Kroenke et al. (25), consists of 9 items, scored from 0 to 3, with a maximum score of 27. Depression is rated 0–4 (Minimal), 5–9 (Mild), 10–14 (Moderate), 15–19 (Moderately severe), and 20–27 (Severe). Depression intensity increases with scores. The PHQ-9 has strong psychometric qualities, with Cronbach’s α coefficients ranging from 0.84 to 0.88, indicating excellent internal consistency. Criterion validity is established by strong connection with Diagnostic and Statistical Manual of Mental Disorders, Fifth Edition (DSM-5) diagnostic criteria (86% sensitivity, 89% specificity). For preliminary screening and depression severity assessment, the measure is useful (26, 27). In high-stress academic environments, PHQ-9 detects depressive symptoms, giving psychological health evidence for curricular improvement in medical education (28).

Spitzer et al.’s GAD-7 has 7 0–3-point items (total score: 0–21). Four anxiety severity levels are 0–4 (Minimal), 5–9 (Mild), 10–14 (Moderate), and 15–21 (Severe) (29). Anxiety severity increases with score. The GAD-7 has strong internal consistency, with a Cronbach’s α coefficient of roughly 0.92, indicating excellent psychometric qualities. Diagnostic validity in clinical and non-clinical groups is 89% sensitivity and 82% specificity, making it ideal for fast anxiety assessment (29, 30). GAD-7 accurately measures anxiety in high-stress populations like medical students and can determine academic stress-related psychological health disorders (30).

Both the PHQ-9 and GAD-7 scales were measured at two time points: 1 week prior to the start of the course (baseline) and 1 week after the completion of the course.

The CEQ comprises 24 items across five dimensions: good teaching (6 items), generic skills (6 items), clear goals (4 items), appropriate workload (4 items), and appropriate assessment (4 items). Items are scored 1–5 points (total score: 120), with higher scores indicating greater course satisfaction (31). The CEQ is widely utilized in higher education with high reliability (Cronbach’s α = 0.87–0.93), demonstrating excellent internal consistency. Both construct and content validity have been validated across multiple international educational contexts, effectively measuring students’ perceptions of teaching quality (31, 32). The CEQ exhibits high sensitivity to teaching methods, curriculum design, and assessment systems, making it particularly suitable for evaluating the impact of pedagogical reforms on student learning experiences (32).

### 2.5 Statistical analysis

All data were analyzed with IBM SPSS 27.0. The Shapiro-Wilk test was used to test the normality of continuous variables. For academic performance indicators (class performance, practical performance, theoretical exam scores, and overall grades), baseline GAD-7 scores, and baseline PHQ-9 scores, the data were non-normally distributed and presented as median (P25, P75). For multi-group comparisons, the Kruskal-Wallis H test was used, and when statistically significant differences were found (*P* < 0.05), *post-hoc* multiple comparisons with Bonferroni correction were performed (*P* < 0.0167). For comparisons between experimental groups and the historical control group, the Mann-Whitney U test was used. For post-course PHQ-9, GAD-7, and CEQ scales, the total scores were normally or approximately normally distributed and presented as mean ± standard deviation. One-way ANOVA with Bonferroni-corrected *post-hoc* multiple comparisons was used for comparisons among the four groups. In addition, the severity levels of depression and anxiety were categorized based on the established criteria of each scale, while gender distribution was analyzed using frequency analysis and the chi-square test. Statistical significance was determined at α = 0.05 (two-tailed).

## 3 Results

### 3.1 Academic performance between experimental groups and historical control group

All experimental groups (Groups B, C, and D) showed significantly lower practical performance (all *P* < 0.001) compared to group A. The median results for groups B, C, and D were 5.17, 5.00, and 3.43 points lower than group A. Table 4 shows group medians and interquartile ranges. No statistically significant differences were observed in class performance, theoretical examination scores, or overall course grades between the experimental and historical control groups (all *P* > 0.05).

**TABLE 4:** Comparison of academic performance between experimental groups and historical control group.

Variable	Group		N		Median (P25, P75)		95% CI of median difference	Mann-Whitney U test	
	Control	Experimental	Control	Experimental	Control	Experimental		*Z*	*P*
Class performance	Group A	Group B	24	22	97.00 (91.00, 97.75)	95.00 (94.00, 96.00)	1.00 (–2.00, 3.00)	–1.219	0.223
		Group C		19		92.00 (92.00, 94.00)	3.00 (0, 5.00)	–1.887	0.059
		Group D		23		92.00 (92.00, 95.00)	2.00 (–1.00, 5.00)	–1.194	0.233
Practical performance	Group A	Group B	24	22	95.00 (93.00, 98.75)^***^	89.92 (87.96, 91.33)	5.17 (3.67, 7.00)	–5.108	0.000
		Group C		19		90.00 (89.00, 92.00)	5.00 (3.00, 7.00)	–4.978	0.000
		Group D		23		91.29 (90.57, 91.57)	3.43 (2.43, 4.86)	–5.289	0.000
Theoretical score	Group A	Group B	24	22	79.50 (73.00, 87.00)	80.00 (74.00, 84.00)	0 (–4.00, 4.00)	–0.077	0.939
		Group C		19		77.00 (73.00, 81.00)	2.00 (–3.00, 7.00)	–0.809	0.419
		Group D		23		79.00 (77.00, 82.00)	1.00 (–4.00, 6.00)	–0.480	0.361
Overall grade	Group A	Group B	24	22	87.00 (81.00, 90.00)	85.50 (82.75, 88.00)	0 (–3.00, 3.00)	–0.022	0.982
		Group C		19		84.00 (82.00, 87.00)	1.00 (–2.00, 5.00)	–0.699	0.484
		Group D		23		86.00 (84.00, 87.00)	1.00 (–3.00, 3.00)	–0.438	0.661

Data are presented as median (P25, P75). Between-group comparisons for non-normally distributed data were performed using the Mann-Whitney U test.

***P < 0.001 indicates statistically significant differences between Group A and the experimental groups (Groups B, C, and D).

### 3.2 Academic performance among experimental groups

Comparative analysis of the three experimental groups indicated significant disparities in class performance scores (*H* = 13.951, *P* = 0.001) and practical performance (*H* = 8.959, *P* = 0.011). *Post-hoc* analysis revealed that groups C and D exhibited considerably inferior class performance compared to group B (*P* < 0.05), however group D demonstrated significantly superior practical performance relative to group B (*P* < 0.05). No statistically significant differences were detected among the three experimental groups either theoretical scores or overall grades (*P* > 0.05). Details are provided in Table 5.

**TABLE 5:** Comparison of academic performance across three experimental groups [M(P25, P75)].

Variable	Group			*H*	*P*
	Group B	Group C	Group D		
Class performance	95.00 (94.00, 96.00)	92.00 (92.00, 94.00)^a^	92.00 (92.00, 95.00)^a^	13.951	0.001
Practical performance	89.92 (87.96, 91.33)	90.00 (89.00, 92.00)	91.29 (90.57, 91.57)^a^	8.959	0.011
Theoretical score	80.00 (74.00, 84.00)	77.00 (73.00, 81.00)	79.00 (77.00, 82.00)	1.537	0.464
Overall grade	85.50 (82.75, 88.00)	84.00 (82.00, 87.00)	86.00 (84.00, 87.00)	2.029	0.363

*^a^*Indicates significant difference from Group B at *P* < 0.05 after multiple comparison correction using Bonferroni method.

### 3.3 Depression and anxiety status

**FIGURE 1:**
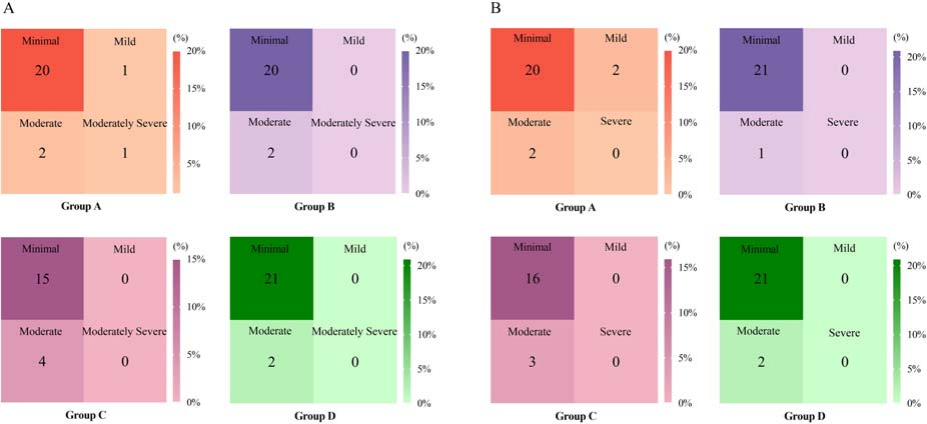
Psychological status. **(A)** Depression status (*n* = 88). **(B)** Anxiety status (*n* = 88).

The distribution of post-course depression levels across groups is presented in Figure 1. In group A, 4 students exhibited varying degrees of depressive symptoms (1 with moderate-severe, 1 with moderate, and 2 with mild depression), while the remaining 20 students were minimal. Groups B, C, and D showed no moderate-severe or moderate depression cases, with only mild depression observed (2 in group B, 4 in group C, and 2 in group D); all other students remained minimal. Regarding anxiety, group A had 4 students reporting anxiety symptoms (2 moderate and 2 mild), with 20 students showing minimal symptoms. Groups B, C, and D exhibited no moderate anxiety cases, reporting only mild anxiety (1 in group B, 3 in group C, and 2 in group D), while all other students scored within the minimal range for anxiety.

### 3.4 Depression, anxiety, and course satisfaction among students of different groups

#### 3.4.1 Depression and anxiety

As shown in Table 6, post-course depression scores were slightly higher in group A compared to the three experimental groups. The historical control group A showed a depression score of 4.71 ± 3.96, while the experimental groups recorded 3.32 ± 1.43 (group B), 3.84 ± 1.57 (group C), and 3.39 ± 1.73 (group D). However, no statistically significant differences in overall depression levels were observed among groups (F = 1.565, *P* > 0.05), indicating comparable depression levels between experimental and historical control groups.

**TABLE 6:** Depression and anxiety scores in experimental and historical control groups (M ± SD).

Variable	Group				*F*	*P*	*Post-hoc*
	Group A^a^	Group B^b^	Group C^c^	Group D^d^			
Depression (PHQ-9)	4.71 ± 3.96	3.32 ± 1.43	3.84 ± 1.57	3.39 ± 1.73	1.565	0.204	-
Anxiety (GAD-7)	4.33 ± 3.32	3.05 ± 1.59	2.32 ± 2.77	2.70 ± 1.43	2.965	0.037	a > c^#^

*Post-hoc* multiple comparisons with Bonferroni correction. The superscripts a, b, c, and d represent Groups A, B, C, and D, respectively.

^#^Indicates group A scored significantly higher than group C (*P* = 0.047).

Group A recorded an anxiety score of 4.33 ± 3.32, while experimental groups showed scores of 3.05 ± 1.59 (group B), 2.32 ± 2.77 (group C), and 2.70 ± 1.43 (group D). However, only the differences between group A and group C attained borderline significance (*P* = 0.047). Regarding the trend in scores, group A had the highest level of anxiety, followed by groups B and D, with group C showing the lowest scores.

#### 3.4.2 Course satisfaction

Course satisfaction results are presented in Table 7. The experimental groups demonstrated significantly higher overall course satisfaction compared to the historical control group A (*F* = 11.306, *P* < 0.001). Group D exhibited the highest satisfaction (95.30 ± 6.91), significantly exceeding all other groups (*P* < 0.05). Groups B (87.59 ± 13.94) and C (83.21 ± 12.35) also showed significantly higher satisfaction than group A (75.04 ± 14.09).

**TABLE 7:** Course satisfaction and secondary dimensions scores of experimental and historical control groups (M ± SD).

Variable	Group				*F*	*P*	*Post-hoc*
	Group A^a^	Group B^b^	Group C^c^	Group D^d^			
Course satisfaction	75.04 ± 14.09	87.59 ± 13.94	83.21 ± 12.35	95.30 ± 6.91	11.306***	< 0.001	d > a; b > a; d > c
Good teaching	19.17 ± 5.47	22.27 ± 8.12	21.11 ± 7.37	24.83 ± 2.96	3.374*	0.022	d > a
Generic skills	17.96 ± 6.58	19.82 ± 7.52	18.68 ± 7.55	21.61 ± 2.86	1.438	0.238	-
Clear goals	13.88 ± 3.71	15.95 ± 2.21	15.79 ± 2.07	16.70 ± 1.64	5.151**	0.003	b > a; d > a
Appropriate workload	12.92 ± 2.95	14.55 ± 3.10	13.95 ± 3.08	15.00 ± 2.84	2.129	0.103	-
Appropriate assessment	11.13 ± 4.19	15.00 ± 3.74	13.68 ± 4.87	17.17 ± 1.80	10.521***	< 0.001	d > a; b > a; d > c

***P < 0.001;

**P < 0.01;

*P < 0.05. *Post-hoc* multiple comparisons with Bonferroni correction. The superscripts a, b, c, and d represent Groups A, B, C, and D, respectively. In the *post-hoc* column, “ > ” means “scored significantly higher than” (e.g., “d > a” indicates Group D scored significantly higher than Group A).

Among the secondary dimensions, significant between-group differences were observed in good teaching (*F* = 3.374, *P* < 0.05), clear goals (*F* = 5.151, *P* < 0.01), and appropriate assessment (*F* = 10.521, *P* < 0.001). *Post-hoc* comparisons further showed that group D scored higher than group A in good teaching, clear goals, and appropriate assessment; group B scored higher than group A in clear goals and appropriate assessment; and group D scored higher than group C in appropriate assessment. Across all dimensions and total scores, group D achieved the highest scores, followed by groups B and C, while group A consistently scored the lowest. Although numerical differences existed in generic skills and appropriate workload dimensions, these did not reach statistical significance (*P* > 0.05).

## 4 Discussion

This study, using standardized scales (PHQ-9, GAD-7), found that implementing FA in a manual therapy course was associated with alleviated anxiety symptoms and enhanced course satisfaction, addressing the research gap in existing literature that often focuses on academic outcomes while neglecting psychological benefits. However, contrary to anticipations, the experimental group had markedly inferior practical examination scores relative to the historical control group receiving FPA, a result that contradicts the predominant literature supporting FA.

### 4.1 Learning outcomes

The finding that the experimental groups had inferior practical performance compared to the historical control group contradicts other domestic and international studies. Wang et al. (19) shown in China that perceived academic freedom greatly improved learning autonomy among Chinese medical students via the indirect influences of psychological empowerment and good academic emotions. Yang et al. (33) discovered that an FA model utilizing “Virtual Standardized Patients-Traditional Chinese Medicine (VSP-TCM) + process feedback” in Traditional Chinese Medicine (TCM) internal medicine training courses markedly enhanced students’ medical interviewing skills, clinical judgment, and overall competencies. Kokkiz et al. (34) established that FA significantly improves the clinical operational competence levels of nursing students. A mixed-methods study also revealed that the incorporation of formative clinical skills assessments, accompanied by real-time feedback and instruction, significantly enhanced learning in early-stage medical students (35).

Although extensive literature supports the positive effects of FA, the lower practical performance scores observed in the experimental groups of this study may stem from multiple factors. FPA may demonstrate “grade inflation,” whereby high performance may not accurately represent true proficiency levels. FPA prioritizes procedural operational assessment, which may indicate superficial learning strategies reliant on rote memorization or test-taking skills, with elevated results not necessarily signifying authentic clinical proficiency (36). Conversely, the experimental groups’ revised assessment prioritized real clinical scenarios and practical application, featuring increased task complexity and evaluation criteria that highlighted comprehensive competency over simple procedural accuracy, which may have led to initially lower scores (15). Another contributing factor may be issues related to the accuracy of implementation of FA strategies, including inadequate teacher training, student unfamiliarity with new assessment forms, or time constraints in integrating feedback loops, which could have diminished the effectiveness of FAs, aligning with Yang et al. (20) emphasis on the necessity of robust faculty support. Moreover, peer assessment constituted 50% of the process evaluation scores for the group B, and student peer evaluations may exhibit inadequate rigor or consistency, hence impacting scoring reliability despite previous demonstrations of evaluation protocols and criteria (13).

Our results suggest that a significant alteration in the study—a paradigm shift in practical assessment scoring criteria from the control group to the experimental groups—may have contributed to this outcome rather than a decline in students’ practical skills. The study design altered assessment frequency, formative assessment timing, and task complexity among the three experimental groups, rendering this conclusion plausible although conjectural. The revised scoring criteria for the experimental groups prioritized clinical reasoning, patient interaction, and problem-solving skills, in contrast to TA models that focused on operational completeness and fluency. The revised requirements were more rigorous and precise, encompassing subtle factors such as force intensity or direction, which could influence variations in therapeutic efficacy.

This view is supported by intragroup analysis: under the standardized new scoring criteria, the group D’s students significantly surpassed the group B (*P* < 0.05), indicating a distinct rising trend. The process evaluation for the group B was executed “during the final 40 min of the second laboratory session of each chapter,” suggesting that students were assessed immediately following the instruction of techniques, with minimal opportunity for consolidation or independent practice, possibly resulting in anxiety and mistakes caused by operational unfamiliarity and inadequate understanding (21). The process evaluation for the group D was “scheduled during the initial 40 min of the first laboratory session of the subsequent chapter,” affording students essential opportunities for review, reflection, and post-class practice, thereby enhancing their proficiency and confidence (37). Instructors in groups B to D gained more experience in doing process evaluation, likely enhancing their instructional direction and assessment comments. In contrast, Group D did not include a comprehensive FPA, so the findings can only short-term information and skill retention, making it difficult to estimate long-term knowledge retention and limiting the study’s interpretability. Based on the academic rationale that formative assessment and feedback support learning and guide students toward competency standards (38), and student feedback that multiple formative assessments combined with the FPA created considerable review pressure, Group D eliminated the FPA and increased the FA weighting to 40%. As a result, the final grade became entirely dependent on seven “mini-assessments.” This approach distributed risk, with relatively lower pressure per assessment, thereby possibly reducing performance anxiety (39). This implies that course design, informed by feedback, should correspond with students’ acceptance and proficiency levels (38). Consequently, our findings suggest that FA, when combined with defined, high-quality clinical standards and sufficient preparation time, may be effective in assisting students in fulfilling rigorous clinical requirements, rather than merely improving test scores. Consequently, this seemingly adverse outcome underscores the intricacy of using FA in high-precision skill courses.

### 4.2 Course satisfaction

The delayed evaluation schedule for Group D provided adequate time for practical preparation, offering students increased opportunities for post-class review and independent practice, thereby enhancing their learning confidence and perceived readiness. This group achieved the highest course satisfaction score (95.30 ± 6.91) and highest scores in subdimensions including “good teaching,” “clear goals,” and “appropriate assessment,” suggesting that this model may enhance learning control and providing clear feedback. The group B, as the first to introduce FA, achieved high satisfaction in “clear goals” and “appropriate assessment,” suggesting initial advantages of FA. The “clear goals” results align with Goodwin et al. (40) findings in neuroscience courses, indicating that when FA is highly aligned with comprehensive teaching objectives, it significantly enhances student learning performance (*P* < 0.05) and increases course satisfaction. However, the immediate post-instruction assessment scheduling could be structurally optimized.

The group C introduced clinical problem-oriented comprehensive evaluation, which, while beneficial for developing clinical reasoning abilities, imposed high task complexity and cognitive demands. Students lacked adequate preparation and capacity support, potentially overwhelming some students (14), resulting in relatively low scores in “appropriate assessment” (13.68 ± 4.87) and overall satisfaction (83.21 ± 12.35). The study showed that the group A’s FPA had significantly lower course satisfaction than all three experimental groups, further suggesting the potential value of FA. Intragroup differences suggest that FA requires balancing “comprehensive ability dimension coverage” with “task complexity,” necessitating equilibrium between complexity and feasibility (41, 42). Our findings found that FA strategies do not significantly increase student cognitive burden and may potentially facilitate adaptation to high clinical standards as well as improve satisfaction, consistent with Xu et al. (43) conclusion that process-oriented FA effectively facilitates complex skill learning without significantly increasing student cognitive burden.

### 4.3 Psychological impact

When comparing the post-course PHQ-9 (depression) and GAD-7 (anxiety) scores among the four groups, post-course anxiety scores of the three experimental groups were all lower than those of the control group (Group A), with an overall statistically significant difference (*F* = 2.965, *P* = 0.037); *post-hoc* comparisons revealed that only Group A scored significantly higher than Group C (*P* = 0.047). Regarding depression, no statistically significant difference was observed among the four groups (*F* = 1.565, *P* = 0.204). The experimental groups had numerically lower mean scores compared to the historical control group (3.32–3.84 vs. 4.71); however, this discrepancy was not statistically significant and should not be interpreted as an indication of an intervention effect. These findings are directionally consistent with prior studies, including Miliæ et al. (8) work indicating that supportive learning environments may help diminish anxiety levels among health science students. This study tentatively attributes the possible psychological benefits of FA to its low-risk, iterative feedback framework, which may cultivate a non-punitive learning environment and alleviate performance pressure. This speculative interpretation aligns with the findings of Wang et al. (19) that positive academic emotions may be significant contributors to the psychological advantages of FA.

Another study confirmed that anxiety reduction significantly contributes to satisfaction enhancement (β = 0.32, *P* < 0.05), supporting the “low pressure-high satisfaction-high effectiveness” teaching loop hypothesis (44). Corina Benjet et al. (45) similarly reported that regular feedback significantly alleviates anxiety (GAD-7) and depression levels (PHQ-9). These results support Trapp et al. (46) proposition that multidimensional assessment effectively enhances students’ control over anxiety symptoms and illustrate FAs distinct contribution to fostering good “psychological-academic” cycles. Conversely, the historical control group’s high-risk FPA may exacerbate anxiety and diminish learning engagement and self-esteem recognition.

Students in the experimental group demonstrated typically sound psychological states prior to the study and were not in a notably poor condition. As a result, it was difficult to identify substantial differences based on small changes following the intervention, indicating that these students were able to maintain consistent psychological state throughout the formative assessment process. Furthermore, the lack of substantial depression alleviation in the experimental group suggests that systemic stressors, such as job competition and academic pressure, may necessitate further interventions beyond educational reforms, aligning with Xie et al. (3) findings on academic burnout in Chinese medical students.

### 4.4 Innovation

This research introduces innovations in multiple aspects: In contrast to typical one-time inter-group comparisons (23), the cross-cohort comparison utilized in this study (Groups B, C, and D versus group A) provides insights into the evolution of learning trends across multiple assessment models, employing dynamically customized evaluation strategies for each cohort to fulfill clinical practical needs. We have initially proposed a multidimensional assessment framework encompassing academic performance, psychological states (PHQ-9, GAD-7), and learning satisfaction, exploring the transition from traditional single-outcome orientation focused on academic achievement and standardized testing toward a comprehensive, process-oriented, multidimensional teaching quality evaluation system. Although the applicability and integration advantages of this model for practice-intensive courses require further validation, this approach shows promise in addressing the dimensional fragmentation issues identified in previous research.

### 4.5 Study limitations

This research also contains multiple limitations. First, the limited sample size restricts the applicability of the findings to other universities or fields. Second, this study used a historical control group, ignored contextual variables, and simultaneously modified total assessment frequency, formative assessment timing, task complexity, and peer assessment across the three experimental groups. Therefore, it is difficult to determine which component or combination of components caused the reported results. All experimental groups used the same practical performance assessment rubric, although other components varied. The results should be seen as suggesting the teaching reform strategy’s overall efficacy rather than any specific element’s causal impact. Third, external stresses, like employment competition and personal factors influencing mental health outcomes, were inadequately addressed. Fourth, despite the utilization of proven PHQ-9 and GAD-7 scales for mental health evaluation, subjective reporting bias may still affect the outcomes. Fifth, this study exclusively assessed results before and after the specific course, without longitudinally tracking the same students throughout various courses within the curriculum. Future research should focus on enlarging the sample size to encompass comparable provincial institutions for the purpose of validating generalizability, implementing longitudinal assessments across multiple courses or tracking changes in graduates’ clinical competency, and exploring critical factors influencing variations in practical performance, such as the relevance of evaluation criteria.

### 4.6 Theoretical implications and practical recommendations

Based on empirical data and clinical teaching experience, this study proposes the following recommendations for implementing FA in PT courses with high practical demands: Design formative tasks aligned with students’ skill levels, and for complex tasks such as problem-oriented assessments, instructors should provide syllabi to balance evaluation complexity (14); strategically configure the frequency and weighting of assessments, advocating a “high-frequency, low-stakes” principle (47); integrate FA with psychological counseling or stress management programs by incorporating mental health support mechanisms to address systemic stressors (3); enhance professional training for instructors on FA (48); and conduct longitudinal studies to track long-term effects, evaluating the sustained impact of FA on clinical practice and professional development. These recommendations may serve as a reference for the application of FA in health science education.

## 5 Conclusion

By prioritizing psychological wellbeing alongside academic achievements, FA in manual therapy courses was associated with reduced anxiety and improved course satisfaction, addressing crucial gaps in TA methods. FA may support skill consolidation and learning control when combined with adequate preparation time and high-frequency, low-stakes assessments, despite lower practical performance scores in experimental groups. The finding likely reflects an evolution toward rigorous, clinically oriented evaluation criteria rather than a decline in skill mastery. These findings suggest that FA may help establish supportive, process-oriented learning environments in high-demand PT education.

## Data Availability

The original contributions presented in this study are included in this article/Supplementary material, further inquiries can be directed to the corresponding authors.
