# Supplementary material for: Longitudinal formative assessment reforms in manual therapy education: impacts on psychological wellbeing and course satisfaction
Source: Front Med (Lausanne). 2026 Jun 26;13:1823631. doi: 10.3389/fmed.2026.1823631 (PMC13350192; doi:10.3389/fmed.2026.1823631)
Supplement: Supplementary file 1 [file Table_1.docx]

**Appendix 1**

**Practical Assessment Scoring Criteria for Group A (2019 Cohort)**

**Assessment Name:** Manual Therapy End-of-Term Practical Assessment

**Assessment Format:** One-Time Standardized Operational Assessment

**Assessment Duration:** Each student is assigned 2 questions per chapter, totaling 7 chapters and 14 questions. Each question allows approximately 3 minutes.

**Scoring Entity:** Independent scoring by the instructor responsible for the corresponding chapter.

| **Scoring Dimension** | **Weight** | **Scoring Criteria Description (1-10 points, 1 = Very Dissatisfied, 10 = Very Satisfied)** |
| --- | --- | --- |
| **Completeness of Operation Process** | 40% | Focuses on evaluating whether the operation steps are complete and in the correct order. Must strictly follow the demonstrated teaching process without omitting steps or making sequence errors. |
| **Proficiency and Fluency** | 30% | Evaluates whether the operation movements are proficient, coherent, and natural. No obvious pauses, hesitations, or unnecessary repetitive actions. |
| **Mastery of Technique Key Points** | 20% | Evaluates the mastery of technique. Must generally meet the standards taught in class. |
| **Overall Impression and Professional Attitude** | 10% | Evaluates appearance, confidence, focus during the operation, and basic respect for the simulated patient. |
