# Supplementary material for: Longitudinal formative assessment reforms in manual therapy education: impacts on psychological wellbeing and course satisfaction
Source: Front Med (Lausanne). 2026 Jun 26;13:1823631. doi: 10.3389/fmed.2026.1823631 (PMC13350192; doi:10.3389/fmed.2026.1823631)
Supplement: Supplementary file 2 [file Table_2.doc]

**Appendix 2**

**Practical Assessment Scoring Criteria for Group B (2020 Cohort)**

**Assessment Name:** Manual Therapy End-of-Term Practical Assessment and Formative Assessment

**Assessment Format:**

Final Practical Assessment (FPA): One-time standardized operational assessment

Formative Assessment (FA): Phase-based evaluations across 7 chapters

**Assessment Duration:**

**FPA:** 2 questions randomly selected per chapter from a predefined pool (5 questions per chapter), approximately 3 minutes per question

**FA:** 40 minutes at the end of the second practical session of each chapter, assessing 6–7 students per session

**Scoring Entity:**

**FPA:** Independent scoring by the instructor responsible for the corresponding chapter

**FA:** Weighted average of evaluations by other students and two instructors

**Practical Assessment Scoring Criteria for Group C (2021 Cohort)**

**Assessment Name:** Manual Therapy End-of-Term Practical Assessment and Clinically Oriented Comprehensive Evaluation

**Assessment Format:**

Retains the FPA and FA structure of Experimental Group B

Introduces a clinically oriented comprehensive evaluation focused on complex clinical scenarios

**Assessment Duration:**

FPA and FA components consistent with Experimental Group B

**Scoring Entity:**

FPA and FA: Same standards as Experimental Group B

**Practical Assessment Scoring Criteria for Group D (2022 Cohort)**

Assessment Name: Manual Therapy Formative Assessment (FA)

**Assessment Format:**

FA-only model (FPA eliminated)

Assessment questions aligned with Experimental Group B

FA conducted in the first 40 minutes of the subsequent chapter’s practical session

**Assessment Duration:**

40 minutes per chapter, administered at the beginning of the next chapter’s practical session

**Scoring Entity:**

Independent scoring by the instructor of the corresponding chapter, following the standards of Experimental Group B

**Scoring Entity:** Independent scoring by the teaching instructor

**Scoring Assessment Table: Joint Mobilization (Example)**

| Scoring for  each section ranges from 1-10 points,  with 1 being very dissatisfied and 10 being very satisfied. | Student ID | |
| --- | --- | --- |
|  | Group | |
|  | Therapist Posture  (10%, including posture changes during assessment and treatment) | |
|  | Patient Posture  (10%, including posture changes during assessment and treatment) | |
|  | communication skills (10%) | |
|  | Manual Technique Operation (50% total, with each subsection accounting for 10%) | Location (fixed or operational part) |
|  |  | Force (magnitude and direction) |
|  |  | Frequency |
|  |  | Duration |
|  |  | Repetitions |
|  | Medical Professionalism (respect for the patient, care for the patient, protection of patient privacy, etc.) (20%) | |
|  | Question Response | |

Notes: The assessment scoring criteria are consistent across all three experimental groups.
